# Effect of positive airway pressure compliance on laryngopharyngeal reflux in obstructive sleep apnea patients

**DOI:** 10.1186/s13104-023-06390-3

**Published:** 2023-06-27

**Authors:** Krongthong Tawaranurak, Methini Werathammo

**Affiliations:** Department of Otolaryngology Head and Neck Surgery, Faculty of Medicine, Prince of Songkla University, Hat Yai, 90110 Songkhla Thailand

**Keywords:** Continuous positive airway pressure, Compliance, Extraesophageal reflux, Laryngopharyngeal reflux, Obstructive sleep apnea

## Abstract

**Objectives:**

To determine the effect of continuous positive airway pressure (CPAP) compliance on symptoms and signs of laryngopharyngeal reflux (LPR) in patients with obstructive sleep apnea (OSA).

**Results:**

Thirty patients were included. The participants were divided into good compliance (n = 21) and poor compliance (n = 9) groups. After 6-month CPAP treatment, the reflux symptom index score significantly decreased in both the good compliance group (20 (17,24) vs. 14 (10,18), p < 0.001) and the poor compliance group (21 (18,25) vs. 10 (5,16), p < 0.05). Reflux finding score was significantly reduced in both the good compliance group (8 (6,9) vs. 4 (3,5), p < 0.001) and the poor compliance group (6 (4,8) vs. 3 (2,4), p < 0.05). However, there were no statistically significant differences between the groups. CPAP treatment reduces the symptoms and signs of LPR. However, CPAP compliance does not correlate with improvement in LPR in patients with OSA.

**Supplementary Information:**

The online version contains supplementary material available at 10.1186/s13104-023-06390-3.

## Introduction

Obstructive sleep apnea (OSA) is a common sleep disorder in middle-aged adults that is characterized by partial or complete obstruction of the upper airway while sleeping. Recurrent episodes of hypoxemia, hypercapnia, and arousal following breathing disturbance lead to poor sleep quality and sympathetic hyperactivity [1, 2]. Patients may present with excessive sleepiness, poor daytime performance or work errors [1]. Moreover, untreated sleep apnea has the relationship with serious health conditions including cardiovascular diseases, metabolic diseases or stroke. These consequences result in increasing healthcare costs [3–5]. Continuous positive airway pressure (CPAP) is the mainstay of treatment for moderate-to-severe OSA [6–8].

Laryngopharyngeal reflux (LPR) is a disease that results from the retrograde flow of gastric contents into the upper airways, particularly the larynx and hypopharynx. Patients typically suffer from hoarseness, chronic cough, throat clearing and globus sensation [9]. Laryngeal examination typically reveals redness, thickening, and edema of the posterior larynx. LPR is usually diagnosed based on a patient’s symptoms and signs of laryngeal irritation. In many cases, no testing is required to make the diagnosis [10]. Previous studies have reported that LPR occurs more frequently in patients with OSA than in the general population. In the general population, the prevalence of LPR is 4–10% whereas the prevalence ranges from 40.3 to 64.3% in OSA patients [9–12]. However, the relationship between LPR and OSA remains unclear. Many studies have shown that CPAP has advantages for LPR symptoms [13–16]. One study noted a positive result for nighttime reflux symptoms after using CPAP [17]. However, the effect of CPAP compliance on LPR has not been investigated. This study aimed to evaluate the improvement in LPR symptoms and signs after CPAP treatment by considering CPAP compliance.

## Materials and methods

### Study Population

This prospective study was performed at the snoring clinic of the Otolaryngology Outpatient Department of Songklanagarind Hospital between August 2019 and January 2022. The study was approved by the Institutional Review Board of the Faculty of Medicine Prince of Songkla University (REC 62-138-13-1). The patients provided informed consent prior to participating in the study and were able to withdraw at any time. Patients newly diagnosed with moderate-to-severe obstructive sleep apnea who received fixed-pressure CPAP therapy (Philips Respironics REMstar Pro), and newly diagnosed LPR by reflux symptom index (RSI) > 13 or reflux finding score (RFS) > 7 were included. Patients taking proton pump inhibitors or H_2_ blockers within 30 days, those with previous esophageal or gastric surgery, those with chronic lung disease, or those who were pregnant were excluded. Demographic and polysomnographic data including age, body mass index (BMI), neck circumference (NC), Epworth sleepiness scale (ESS) [18, 19], apnea-hypopnea index (AHI), lowest oxygen saturation, RSI, and RFS were collected at baseline. CPAP compliance, RSI, and RFS were evaluated after 6 months of CPAP treatment.

### RSI questionnaire and RFS

The RSI is a nine-item self-administered questionnaire with scores ranging from 0 to 45 [20]. RFS is an eight-item clinical severity rating scale based on endoscopic findings with scores ranging from 0 to 26 [21]. Endoscopic examination and evaluation of the larynx were performed by the same otolaryngologist (K.W.) at baseline and 6 months before loading the CPAP usage data. An RSI of > 13 or an RFS of > 7 is considered indicative of LPR [20, 21].

### CPAP compliance

CPAP compliance was objectively measured by the SD card recording of nightly use over 6 months. Data were collected regarding mean CPAP pressure use (cmH_2_O), average usage (hours/day), and mean percentage of usage for > 4 h/night. Good CPAP compliance is defined as the use of CPAP therapy for an average of 4 h each night for 70% of the nights [22].

### Sleep studies

Standard sleep study (Compumedics E series, Compumedics) was performed in the sleep laboratory with an attended sleep technician, including electroencephalography, electrooculography, electromyography, electrocardiography, thermistors, nasal pressure transducers, chest and abdominal belts, and pulse oximetry. Polysomnographic data were scored manually using standard criteria [23]. Apnea was scored as a decrease in the thermistor signal ≥ 90% of the pre-event baseline for ≥ 10 s [24]. Hypopnea was defined as a 30% decrease in nasal pressure signals for ≥ 10 s, associated with ≥ 3% desaturation or arousal [24]. The AHI was measured by the number of apnea and hypopnea events per total sleep hour. An AHI score of 15–30 events/hour was considered moderate OSA and an AHI score of > 30 events/h was considered severe OSA [6].

### Statistical analysis

Baseline data were reported as number (percentage), mean (standard deviation), or median (interquartile range). Comparisons between groups were performed using the chi-squared test or Fisher’s exact test for nominal variables, Wilcoxon–Mann–Whitney U test for ordinal variables with abnormal distribution, and t-test for ordinal variables with normal distribution. The linear mixed-effects model was used to compare data within the same groups at baseline and after 6 months of CPAP treatment. All statistical analyses were performed using R software (version 4.0.2). A p value of < 0.05 was considered statistically significant.

## Results

All patients who entered the protocol (n = 30) completed the study. The patients were divided into two groups: 21 patients in the good compliance group and nine patients in the poor compliance group. The mean age was 51.6 ± 12.5 years in the good compliance group and 49.0 ± 9.0 years in the poor compliance group. The average BMI was 30.0 ± 5.3 kg/m^2^ in the good compliance group and 33.6 ± 7.0 kg/m^2^ in the poor compliance group (p = 0.133). The severity of OSA and lowest oxygen saturation were not significantly different between the groups. Moreover, the baseline RSI and RFS were similar in both the groups (Table 1).

**Table 1:** Baseline characteristics of OSA patients (n = 30)

Characteristic	Good compliance group (n = 21)	Poor compliance group (n = 9)	p value
Age (years) mean (SD)	51.6 (12.5)	49.0 (9.0)	0.583
Gender Male, n (%) Female, n (%)	16 (76.2) 5 (23.8)	5 (55.6) 4 (44.4)	0.389
BMI (kg/m^2^), mean (SD)	30.0 (5.3)	33.6 (7.0)	0.133
NC (cm), mean (SD)	39.4 (3.0)	38.6 (4.1)	0.519
ESS, mean (IQR)	11 (10,16)	10 (10,11)	0.686
AHI (events/hour), mean (SD)	49.2 (26.8)	66.4 (31.4)	0.137
Lowest oxygen saturation (%), mean (SD)	76.0 (11.8)	72.9 (12.3)	0.512
RSI, median (IQR)	17 (14,22)	21 (18,22)	0.194
RFS, median (IQR)	8 (5,10)	6 (4,7)	0.097

Abbreviations: OSA, obstructive sleep apnea; BMI, body mass index; NC, neck circumference; ESS, Epworth Sleepiness Scale; AHI, apnea–hypopnea index; RSI, Reflux symptom index; RFS, Reflux finding score; SD, standard deviation; IQR, interquartile range

At 6 months of CPAP treatment, the mean CPAP pressure was 8.0 ± 2.0 cmH_2_O in the good compliance group, compared with 8.7 ± 3.1 cmH_2_O in the poor compliance group (p = 0.468). In the good compliance group, the average percentage of CPAP usage for > 4 h/night was 88.4 ± 8.3% and the average nightly use was 6.5 (5.6,7.2) hours. In the poor compliance group, the average percentage of CPAP usage for > 4 h/night was 50.7 ± 12.5% and the average nightly use was 3.6 (3.3,5.2) hours.

**Fig. 1:**
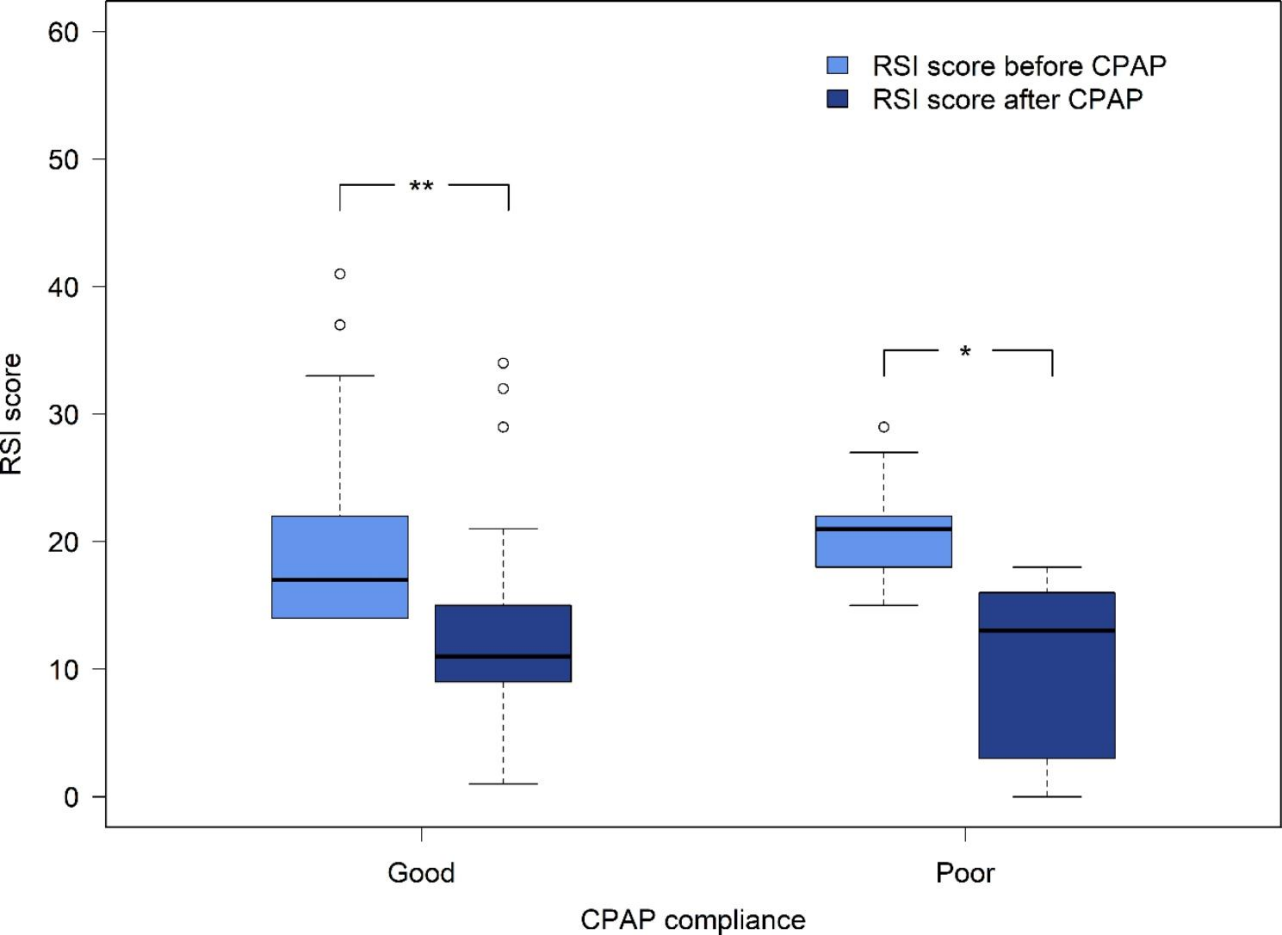
Comparison of RSI score at before and after 6 months CPAP treatment. *p < 0.05 **p < 0.001

**Fig. 2:**
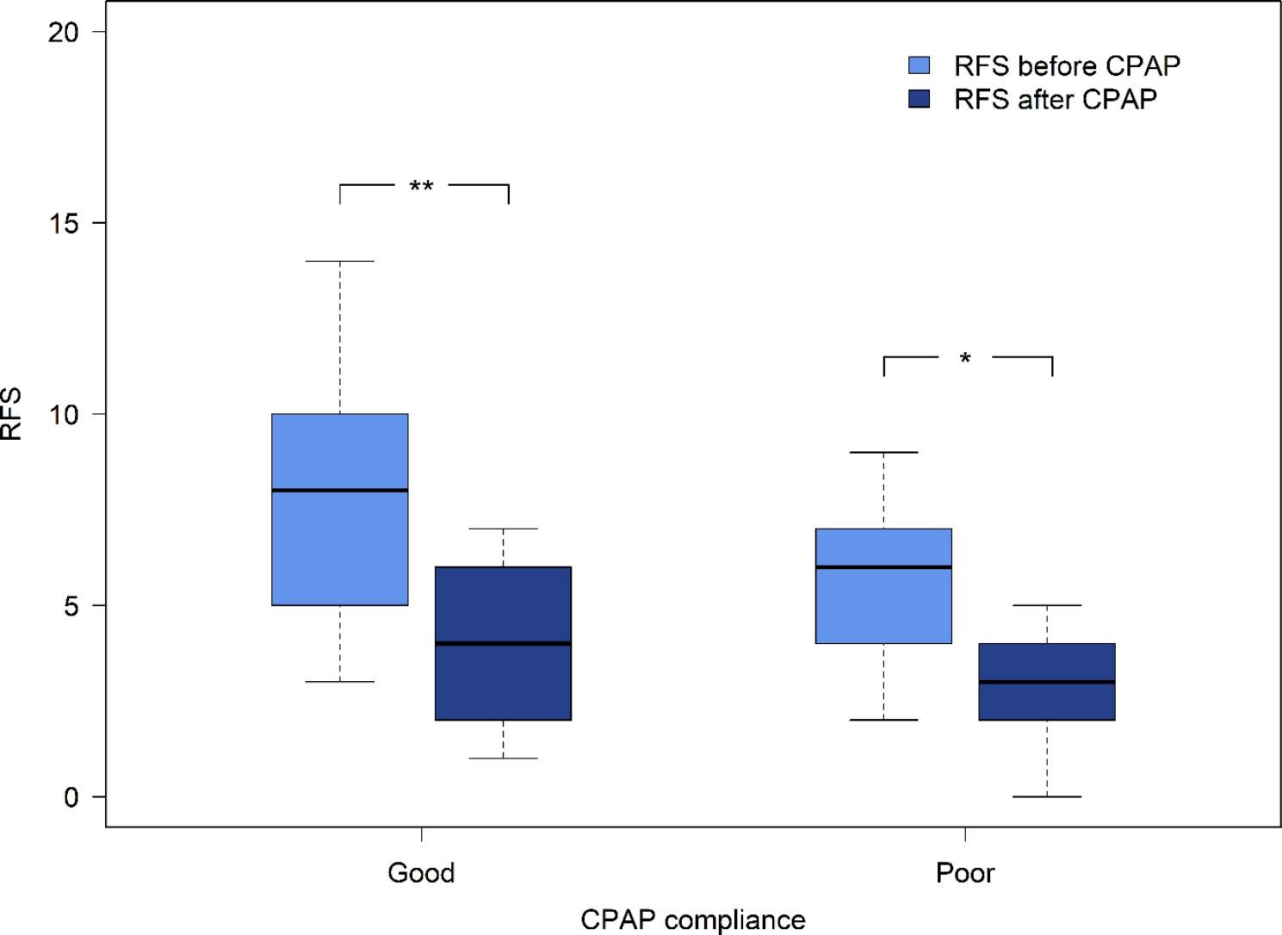
Comparison of RFS at before and after 6 months CPAP treatment. *p < 0.05 **p < 0.001

The RSI score significantly decreased after 6 months of CPAP treatment in the good compliance group (20 (17,24) vs. 14 (10,18), p < 0.001) and the poor compliance group (21 (18,25) vs. 10 (5,16), p < 0.05) (Fig. 1). However, there was no statistically significant difference between the two groups (p = 0.066). In the good compliance group, RFS was significantly reduced after 6 months of CPAP therapy (8 (6,9) vs. 4 (3,5), p < 0.001), similar to the poor compliance group (6 (4,8) vs. 3 (2,4), p < 0.05) (Fig. 2). However, there was no statistically significant difference between the two groups (p = 0.456). The mean differences in the RSI and RFS between the two groups were also analyzed. RSI score differences were 6.6 ± 4.6 and 11 ± 8.0 in the good compliance and poor compliance groups, respectively (Additional file 1). For the good compliance group, the mean RFS difference was 3.6 ± 2.9, compared with 2.8 ± 1.7 in poor compliance group (Additional file 2). There were no significant differences between the two groups in terms of RSI score (p = 0.066) or RFS (p = 0.456).

At 6 months of CPAP treatment, the ESS score significantly decreased in both groups (p = 0.001 in the good compliance group vs. p = 0.018 in the poor compliance group). There were no significant changes in BMI (p = 0.274 in the good compliance group vs. p = 0.099 in the poor compliance group) or NC (p = 0.329 in the good compliance group vs. p = 0.139 in the poor compliance group) in both groups.

There was a relatively strong positive correlation between the difference in BMI and the RSI score after the 6 months of CPAP treatment (r = 0.042, p = 0.02). However, there was no correlation between the differences in BMI and RFS scores (r = 0.07, p = 0.720). (Additional file 3)

## Discussion

A high incidence of LPR in OSA patients has been reported in many studies, but the mechanism remains unclear [9, 12]. One proposed mechanism is that OSA has a direct effect on LPR by increasing transdiaphragmatic pressure and decreasing intrathoracic pressure during apneic events, resulting in an increased risk of microaspiration of gastric content to the pharynx [25]. Another mechanism may be the transient loss of protective reflexes of the upper esophageal sphincter during sleep [26]. Many studies have confirmed that LPR and OSA may have similar risk factors, such as male sex, age, alcohol intake and high BMI [9, 16, 27]. In our study, most patients were male and the mean BMI was relatively high in both groups.

BMI is a marker of obesity and a risk factor for LPR. A previous study reported that an increase in BMI is associated with a greater risk of developing more severe reflux symptoms [28]. Weight loss can lead to the resolution of reflux disease and apnea symptoms [29]. In our study, no correlation was found between BMI and reflux symptoms or signs at the beginning. However, we found a strong positive correlation between changes in BMI and RSI after 6-month CPAP use. Thus, weight change is likely to be an important factor that changes overtime and influences the improvement of LPR symptoms.

The correlation between OSA severity and LPR remains controversial. Elhennawi et al. reported that patients with severe OSA had significantly higher nocturnal LPR reflux episodes than those with mild disease [30]. In contrast, Lee et al. reported that OSA severity was not related to the severity of LPR parameters [31]. In our study, most OSA patients with LPR symptoms had severe OSA (80.7%), with a mean AHI of 53.8 events/hour. However, we found that OSA severity based on AHI did not correlate with RSI and RFS parameters. Moreover, changes in AHI were not correlated with LPR symptoms.

The use of CPAP can reduce a patient’s arousal and movement during sleep, which can prevent changes in abdominal pressure, and thus reduce reflux events. In our study, CPAP treatment, regardless of compliance, helped reduce LPR symptoms after 6 months of use. These results are consistent with those of Eryilmaz et al., who demonstrated that CPAP therapy in severe OSA significantly improved the subjective reflux at 3 months [13]. Literature regarding CPAP compliance remains unclear about the nightly duration of CPAP usage required to normalize functional status, increase memory, and decrease the incidence of cardiovascular events, including hypertension, which ranges from a minimum of 4 to 6–8 h/night across studies. However, there is a relationship between CPAP adherence and improvements in health and quality of life. Four h/night for 70% of monitoring nights is typically used in studies to differentiate between CPAP adherence and nonadherence [22, 32–37]. In our study, the poor compliance group showed improvement in LPR parameters, similar to that of the good compliance group. The relatively high percentage of compliance (approximately 50%) with a mean usage of 3.6 h/night might be the reason for this improvement. Tamanna et al. reported that a minimum CPAP compliance of 25% would improve nocturnal gastroesophageal reflux [17]. Moreover, all patients in our study used the fixed pressure mode of the CPAP device, and the average pressure was not too high, approximately 8 cmH_2_O, which cannot cause CPAP-induced aerophagy which may worsen gastroesophageal reflux disease [38, 39].

One strength of our study is that it is the first study assessing the effect of CPAP compliance, in terms of good vs. poor adherence by using 4 h/night and ≥ 70% of nights as a cut-off point, in evaluating improvements of LPR symptoms and signs. Moreover, we followed patients for 6 months without anti-reflux medication prescriptions.

### Limitations

Limitations of this study include the small number of patients and the limited investigation of LPR. To clarify these results, a larger study with more patients and long-term follow-up is needed, and additional studies using objective measurements of LPR are warranted.

## Conclusion

CPAP treatment improves LPR symptoms and laryngeal findings in patients with OSA. The effect of CPAP compliance in terms of good or poor compliance may not correlate with improvement in LPR symptoms. Thus, OSA patients with LPR symptoms should be encouraged to undergo CPAP therapy and risk factor modification before clinicians prescribe antireflux medication.

## Electronic supplementary material

Below is the link to the electronic supplementary material.

Supplementary Material 1

Supplementary Material 2

Supplementary Material 3

## Data Availability

All data are presented in the manuscript.

## List of abbreviations

CPAP: Continuous positive airway pressure
LPR: Laryngopharyngeal reflux
OSA: Obstructive sleep apnea
RSI: Reflux symptom index
RFS: Reflux finding score
BMI: Body mass index
NC: Neck circumference
ESS: Epworth sleepiness scale
AHI: Apnea-hypopnea index
