# Supplementary figures and images for: Effect of positive airway pressure compliance on laryngopharyngeal reflux in obstructive sleep apnea patients
Source: BMC Res Notes. 2023 Jun 27;16:124. doi: 10.1186/s13104-023-06390-3 (PMC10294299; doi:10.1186/s13104-023-06390-3)

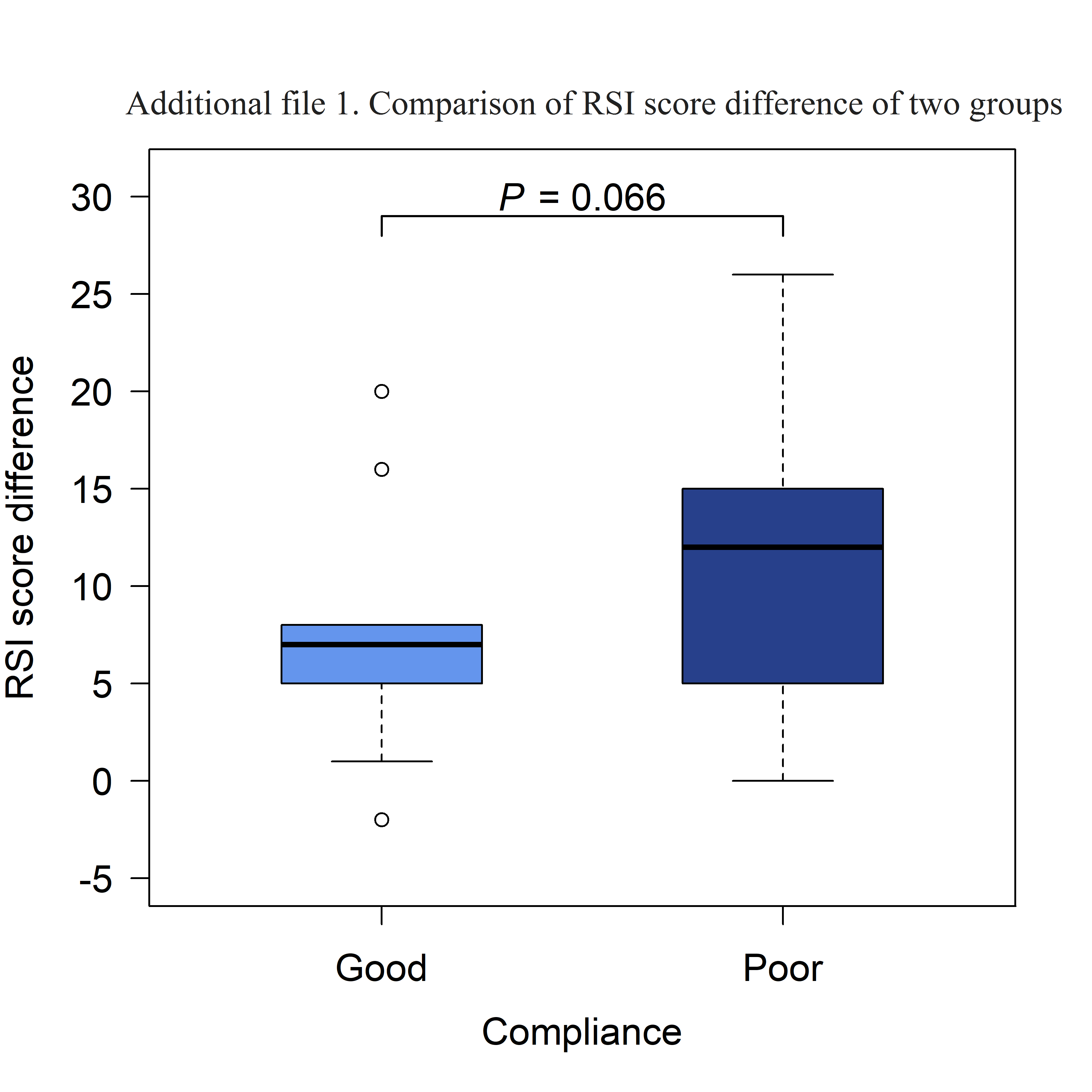

Supplement: Supplementary file 1 — Supplementary Material 1 [file 13104_2023_6390_MOESM1_ESM.tiff]

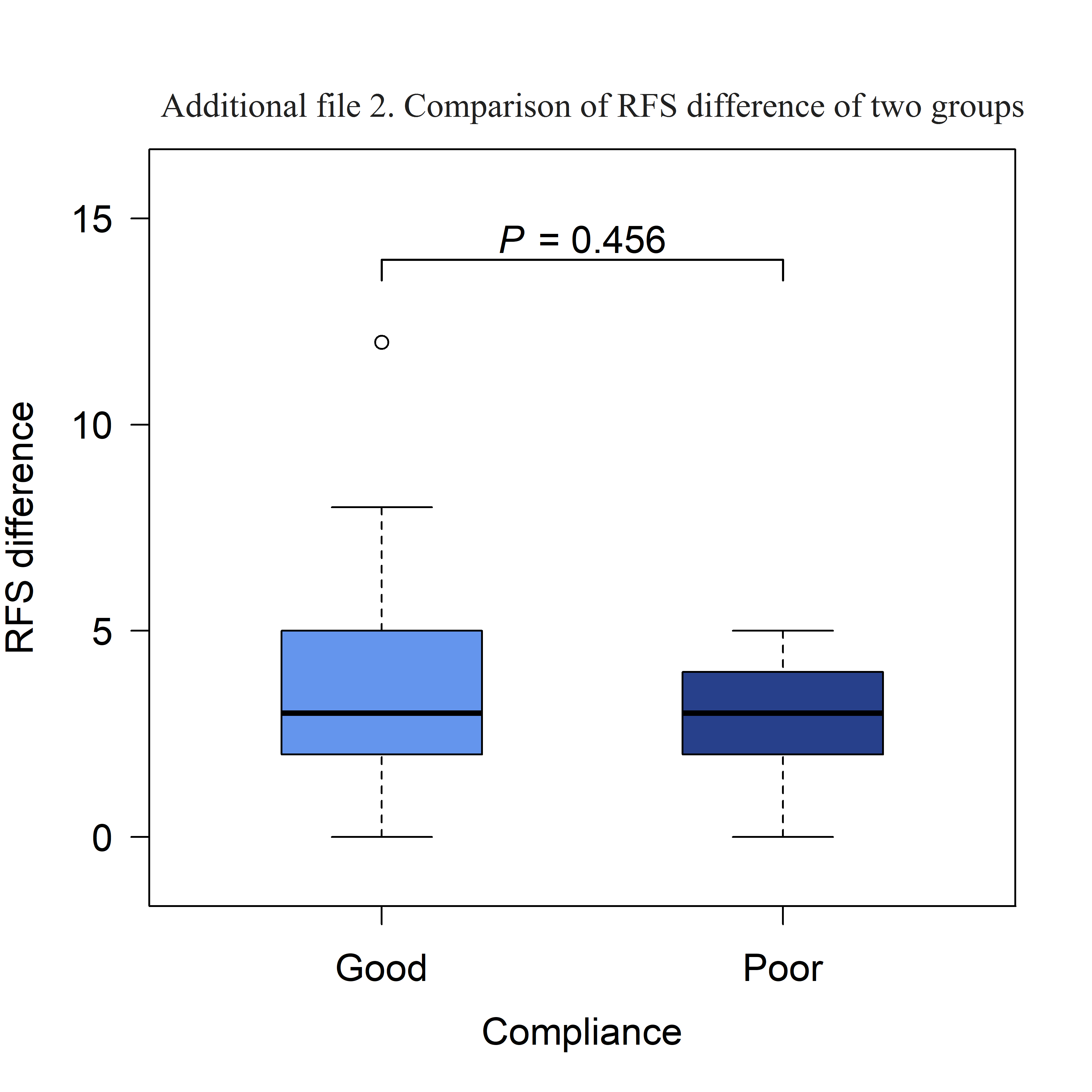

Supplement: Supplementary file 2 — Supplementary Material 2 [file 13104_2023_6390_MOESM2_ESM.tiff]
