# Supplementary material for: Effect of positive airway pressure compliance on laryngopharyngeal reflux in obstructive sleep apnea patients
Source: BMC Res Notes. 2023 Jun 27;16:124. doi: 10.1186/s13104-023-06390-3 (PMC10294299; doi:10.1186/s13104-023-06390-3)
Supplement: Supplementary file 3 — Supplementary Material 3 [file 13104_2023_6390_MOESM3_ESM.docx]

Additional file 3. The correlations between various parameters

| Correlations | r | p value^#^ |
| --- | --- | --- |
| AHI and RSI  AHI and RFS  Difference AHI and difference RSI  Difference AHI and difference RFS  BMI and RSI  BMI and RFS  Difference BMI and difference RSI  Difference BMI and difference RFS | -0.043  0.095  0.090  0.012  -0.040  -0.001  0.422  0.069 | 0.819  0.610  0.637  0.950  0.830  0.996  0.020*  0.718 |

AHI, apnea-hypopnea index; RSI, Reflux symptom index; RFS, Reflux finding score; BMI, Body mass index

#Pearson’s product-moment correlation; *p<0.05
